# Mechanistic Systems Modeling to Improve Understanding and Prediction of Cardiotoxicity Caused by Targeted Cancer Therapeutics

**DOI:** 10.3389/fphys.2017.00651

**Published:** 2017-09-08

**Authors:** Jaehee V. Shim, Bryan Chun, Johan G. C. van Hasselt, Marc R. Birtwistle, Jeffrey J. Saucerman, Eric A. Sobie

**Affiliations:** ^1^Department of Pharmacological Sciences, Icahn School of Medicine at Mount Sinai New York, NY, United States; ^2^Department of Biomedical Engineering, University of Virginia Charlottesville, VA, United States

**Keywords:** tyrosine kinase inhibitors, quantitative systems pharmacology, mathematical modeling, drug-induced adverse events

## Abstract

Tyrosine kinase inhibitors (TKIs) are highly potent cancer therapeutics that have been linked with serious cardiotoxicity, including left ventricular dysfunction, heart failure, and QT prolongation. TKI-induced cardiotoxicity is thought to result from interference with tyrosine kinase activity in cardiomyocytes, where these signaling pathways help to control critical processes such as survival signaling, energy homeostasis, and excitation–contraction coupling. However, mechanistic understanding is limited at present due to the complexities of tyrosine kinase signaling, and the wide range of targets inhibited by TKIs. Here, we review the use of TKIs in cancer and the cardiotoxicities that have been reported, discuss potential mechanisms underlying cardiotoxicity, and describe recent progress in achieving a more systematic understanding of cardiotoxicity via the use of mechanistic models. In particular, we argue that future advances are likely to be enabled by studies that combine large-scale experimental measurements with Quantitative Systems Pharmacology (QSP) models describing biological mechanisms and dynamics. As such approaches have proven extremely valuable for understanding and predicting other drug toxicities, it is likely that QSP modeling can be successfully applied to cardiotoxicity induced by TKIs. We conclude by discussing a potential strategy for integrating genome-wide expression measurements with models, illustrate initial advances in applying this approach to cardiotoxicity, and describe challenges that must be overcome to truly develop a mechanistic and systematic understanding of cardiotoxicity caused by TKIs.

## Introduction

Tyrosine kinase inhibitors (TKIs) constitute a class of cancer therapeutics, many of which are known to cause cardiotoxicity as a major adverse event. Reported cardiotoxicities include heart failure, cardiomyopathy, conduction abnormalities, QT prolongation, and myocardial injury. The most common toxicity is systolic dysfunction or cardiomyopathy, potentially leading to heart failure, which is most likely mediated through direct toxicity of cardiomyocytes (Albini et al., 2010; Eschenhagen et al., 2011; Force and Kolaja, 2011; Raschi and De Ponti, 2012; Ewer and Ewer, 2015).

Trastuzumab, an inhibitor of the HER2 receptor tyrosine kinase (Slamon et al., 2001; Piccart-Gebhart et al., 2005; Romond et al., 2005; Force et al., 2007) was both the first monoclonal antibody TKI given FDA approval (in 1998) and the first TKI reported to cause cardiotoxicity (Wu et al., 2016). Since the reports of trastuzumab-induced toxicity, several additional targeted cancer therapeutics have been classified as cardiotoxic, observations that have contributed to the emergence of a new research field, *cardio-oncology* (Albini et al., 2010; Bellinger et al., 2015).

Previous studies have shown that TKI-related cardiotoxicity, as seen with trastuzumab, is mostly due to the targeting of pathways that are shared between malignancies and cardiovascular cells (De Keulenaer et al., 2010; Bellinger et al., 2015). Investigations of these adverse events revealed that many of the tyrosine kinases targeted by TKIs serve critical roles in survival and maintenance of cardiomyocytes, leading to unintended *on-target* toxicity. At the same time, many TKIs inhibit multiple kinases simultaneously, which can cause *off-target* toxicity (Chen et al., 2008; Force and Kerkelä, 2008; Force and Kolaja, 2011).

Despite the risk of cardiotoxicity, TKIs are still one of the highly effective and favored cancer therapeutics on the market (Eschenhagen et al., 2011; Force and Kolaja, 2011). The success of drugs such as trastuzumab and imatinib, a small molecule inhibitor used to treat chronic myeloid leukemia (CML), has inspired the development of additional TKIs. As of April, 2015, 25 small molecule TKIs have entered the market (Shah and Morganroth, 2015), with many more under development (Bellinger et al., 2015). Given the booming research in the development of TKIs, it would be beneficial to develop a systematic strategy to: (1) evaluate and predict how new TKIs will affect signaling networks in cardiomyocytes; and (2) identify interventions that can reverse and/or mitigate any associated cardiotoxicity. These questions are well-suited to be addressed using a quantitative systems pharmacology (QSP) approach that combines large-scale measurements with mechanism-based mathematical modeling. The diversity of TKI targets and the complexity of cellular mechanisms responsible for cardiotoxicity mean that two drugs with similar targets may operate through different mechanisms, and the effects of two TKIs with different targets may converge on a common pathway. Untangling this type of complexity generally requires computational approaches that are based on biological mechanisms. Therefore, our aims in this Perspective are to review the progress that systems approaches have made in predicting TKI-induced cardiotoxicity and to offer suggestions for how mathematical modeling can be applied to elucidate mechanisms and predict potential adverse events caused by new drugs.

## Tyrosine kinase signaling in cancer and strategies underlying TKIs

The canonical roles of tyrosine kinases are found in mitogenesis and related processes such as differentiation, metabolism, and migration. Constitutive activation of tyrosine kinase (TK) signaling, via either gain-of-function (GOF) mutations or overexpression due to gene amplification, is found in about 70% of malignancies (Blume-Jensen and Hunter, 2001; Chen et al., 2008). Well-understood examples include overexpression of ERBB2 in HER2^+^ breast cancer (Force et al., 2007) and the constitutively active oncogenic fusion protein BCR-ABL, which can cause CML (Force et al., 2007; Chen et al., 2008; Force and Kolaja, 2011). This dependency of tumor formation and proliferation on TK signaling led to the rise of TKIs as promising anti-cancer therapeutics.

**Figure 1:**
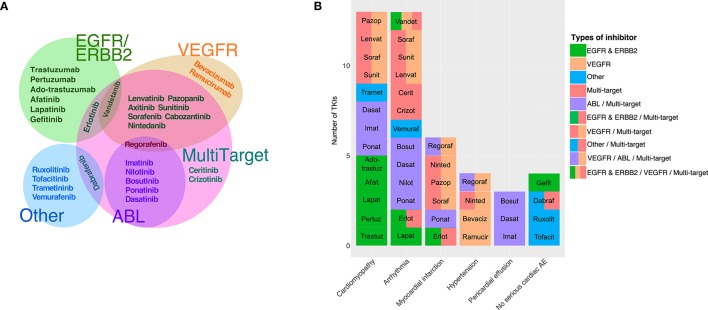
TKI targets and associated adverse events. **(A)** Euler diagram of tyrosine kinase inhibitors grouped based on the primary intended target(s). The three major primary targets are EGFR/ERBB2 (8 TKIs), VEGFR (11 TKIs), and ABL (6 TKIs). The category “Other” comprises five relatively newer TKIs with primary targets in different categories, such as vemurafenib (B-Raf). Out of 30 approved TKIs, 18 were identified as having intended targets in more than one category. **(B)** Black box warnings associated with tyrosine kinase inhibitors are indicated, with closely-related toxicities grouped to ease visualization. *Cardiomyopathy* category includes: “cardiac dysfunction,” “congestive heart failure,” “left ventricular dysfunction,” and “cardiomyopathy.” *Arrhythmia* includes: “prolonged QT interval,” “cardiac bradyarrhythmia,” and “cardiac arrhythmia.” *Pericardial effusion* includes both “pericardial/pleural effusion,” and “cardiac tamponade.” Four approved drugs have no cardiac-associated boxed warning (i.e., no serious cardiac adverse events listed in the drug's package insert).

Currently, there are two chemical classes of TKIs: (1) humanized monoclonal antibodies (mAbs) and (2) small molecule inhibitors (Force et al., 2007; Chen et al., 2008; Force and Kolaja, 2011). Small molecule TKIs can be further subcategorized based on whether they compete with ATP for the binding pocket or interact with other regions of the protein (Force and Kolaja, 2011). Additionally, TKIs are often identified by the intended target(s) or the target specificity (Force et al., 2007; Bellinger et al., 2015; Gharwan and Groninger, 2015). The most common target groups that are used to classify TKIs include EGFR/ERBB2 inhibitors, VEGFR inhibitors, ABL inhibitors, and multi-targeted drugs that are designed to inhibit at least two different target groups such as VEGFR and ABL (see Table 1 for descriptions of the important cellular signaling proteins mentioned in the manuscript). Figure 1A shows the currently-approved TKIs, grouped by the published targets, and indicates how these classifications frequently overlap.

**Table 1:** Biological signaling components potentially relevant to toxicity.

**Common name**	**Full name**	**Description**
EGF	Epidermal growth factor	Extracellular peptide that signals through autocrine and paracrine mechanisms
Neuregulin1	N/A	Extracellular peptide that signals through autocrine and paracrine mechanisms
ERBB2/HER2	Human epidermal growth factor receptor 2	EGFR family receptor tyrosine kinase
ERBB1/EGFR	Receptor for EGF	EGFR family receptor tyrosine kinase
VEGF	Vascular endothelial-derived growth factor	Extracellular peptide that signals through autocrine and paracrine mechanisms
VEGFR	Receptor for VEGF	Receptor tyrosine kinase
PDGF	Platelet-derived growth factor	Extracellular peptide that signals through autocrine and paracrine mechanisms
PDGFR	Receptor for PDGF	Receptor tyrosine kinase
ABL1	Abelson murine leukemia viral oncogene homolog 1	cytoplasmic tyrosine kinase
BCR-ABL	Fusion protein of ABL1 and Breakpoint cluster region protein (BCR)	cytoplasmic fusion tyrosine kinase
Raf-1/c-Raf	N/A	cytoplasmic serine/threonine kinase
ERK	Extracellular signal-related kinase	cytoplasmic serine/threonine kinase
JNK	c-Jun N-terminal kinase	cytoplasmic serine/threonine kinase
PI3K	Phosphatidylinositide 3-kinase	cytoplasmic lipid kinase
Akt/PKB	Also known as Protein Kinase B	cytoplasmic serine/threonine kinase
Src/c-Src	N/A	cytoplasmic tyrosine kinase
AMPK	AMP-activated protein kinase	cytoplasmic serine/threonine kinase

## Reported serious cardiac side effects of TKIs

The initial discovery of TKI-induced cardiotoxicity was made during the groundbreaking clinical trials of trastuzumab, the first such drug to be marketed (Seidman et al., 2002; De Keulenaer et al., 2010). However, estimated cardiotoxicities of 3–7% with trastuzumab alone and 25% when the drug was administered with an anthracycline (Slamon et al., 2001) were only determined during a *post hoc* analysis. Similar retrospective analyses have been performed to estimate that sunitinib causes left ventricular dysfunction with an incidence of 4–11% (Yeh and Bickford, 2009; Lenneman and Sawyer, 2016) and the VEGFR inhibitor bevacizumab induces either cardiomyopathy or heart failure in 1.5–3% of patients (Yeh and Bickford, 2009). These examples demonstrate the difficulties associated with identifying cardiotoxicity during drug development. Because clinical trials are primarily focused on evaluating efficacy, they often lack appropriate safety screening measures to identify side effects (Force et al., 2007).

Overall, of the 30 TKIs currently marketed for use in the United States, 26 list serious cardiac side effects as a “black box warning” in their prescription information (FDA and CDER, 2012; Boehringer Ingelheim International GmbH, 2014; Gharwan and Groninger, 2015). The cardiac related black box warnings of TKIs can be categorized into: cardiomyopathy, arrhythmia, myocardial infarction, hypertension, and pericardial effusion, based on the specific potential adverse events listed in the package insert. In Figure 1B, which indicates both the adverse events and the target class for each TKI, we observe no obvious association between the intended primary target, and the reported cardiac risks.

## Mechanisms underlying cardiotoxicity caused by TKIs

The initial discovery of TKI-induced cardiotoxicity was met with surprise due to the fact that cardiomyocytes are non-dividing and terminally differentiated (Force et al., 2007). Since TKs were mostly known for their role in proliferation and their association with cancer, these kinases were not expected to have any essential role in cardiomyocytes, and toxicity in heart was not anticipated (Chen et al., 2008; Bellinger et al., 2015). The discovery of TKI-induced cardiotoxicity, therefore, became a driving force for uncovering the roles of tyrosine kinases in heart. The research spurred by these adverse events has allowed us to appreciate that many of the pathways responsible for proliferation in malignant cells also play important roles in cardiomyocytes in: (1) survival signaling; (2) mitochondrial and sarcoplasmic reticulum (SR) homeostasis; and (3) electrical and contractile function.

### Survival signaling

The role of tyrosine kinases in cardiomyocyte survival signaling was first discovered through the on-target cardiotoxicity caused by trastuzumab. Before the cardiotoxicity reports, expression of trastuzumab's target, ERBB2, was reported to be low in cardiomyocytes, and this receptor's role was unknown (Bellinger et al., 2015). However, subsequent studies have discovered that ERBB2 plays an important role in maintaining cardiomyocyte health, evidenced by the spontaneous dilated cardiomyopathy that results from ERBB2 knockout (Crone et al., 2002; De Keulenaer et al., 2010). More specifically, ERBB2 in cardiomyocytes has been shown to serve as a co-receptor in a critical cardiomyocyte survival pathway initiated by neuregulin-1 (Mellor et al., 2011; Bellinger et al., 2015). Neuregulin-1, a paracrine factor secreted by cardiac endothelial cells, activates mitogenic pathways through ERBB2 heterodimer formation with other members of the EGFR family, ERBB3 or ERBB4 (Chen et al., 2008; De Keulenaer et al., 2010).

Similarly, another EGFR family member, ERBB1, has also been implicated in cardioprotection and myocyte survival (Mellor et al., 2011; Bellinger et al., 2015), including cardiomyocyte defenses against the deleterious consequences caused by excessive β-adrenergic receptor stimulation (Chen et al., 2008). This role was based on the finding that an ERBB1 inhibitor, erlotinib, exacerbates isoproterenol-induced myocardial injury (Chen et al., 2008). Erlotinib is associated with cardiotoxicity, including cardiac arrhythmia and myocardial infarction (Gharwan and Groninger, 2015). One of the downstream pathways common to signaling through ERBB1 and ERBB2 is the lipid kinase PI3K, which in turn activates the protein kinase Akt. The PI3K-Akt axis is critical in survival signaling, and dysregulation of this pathway has been shown to induce ischemic heart disease, hypertrophy, and heart failure (Reichelt et al., 2017).

Raf-1, which belongs to the Raf family of serine/threonine kinases, is another important component of pro-survival signaling that has been linked to both inherited heart disease (Dhandapany et al., 2014), and TKI-induced cardiotoxicity. Specifically, Raf-1 has been identified as a critical component of cardiotoxicity caused by sorafenib (Force et al., 2007; Chen et al., 2008), a multi-target TKI used to treat renal and liver cancers. The inhibition of Raf-1 by sorafenib is thought to block survival signaling through the protein kinase ERK, and concurrently to disinhibit pro-apoptotic kinases. This dual action of pro-survival signaling inhibition and apoptotic signaling activation can culminate in cell death (Force et al., 2007).

### Sarcoplasmic reticulum and mitochondrial homeostasis

In addition to pro-survival signaling, TKs are known to be closely linked to processes that maintain the health and function of cardiomyocytes through mitochondrial and SR homeostasis (Force and Kolaja, 2011). Mitochondria are responsible for matching the cellular supply of ATP with the energetic demand whereas the SR functions to both modulate the quantity of Ca^2+^ released with each heartbeat and to control the processing of many critical proteins.

When TKI-induced toxicity involves mitochondrial or SR function, the processes seem to be closely linked. Specifically, mitochondrial dysfunction resulting from TKI treatment can lead to membrane permeabilization and the release of reactive oxidative species to the cytoplasm. This oxidative stress can in turn lead to SR dysfunction through both altered Ca^2+^ release and the activation of signaling pathways that may ultimately lead to apoptosis (Groenendyk et al., 2010).

Cardiotoxicity caused by imatinib, a multi-targeted ABL inhibitor, has been proposed to follow this precise mechanism (Kerkelä et al., 2006; Force et al., 2007; Mellor et al., 2011). The on-target effect of imatinib has been linked to the disturbance of SR homeostasis via inhibition of an ABL isoform that is localized in the SR. This can eventually initiate apoptosis through JNK activation. Consistent with this hypothesis, postmortem histological examinations of patients treated with imatinib have revealed dilated SR structures, and experiments in isolated cardiomyocytes shown that imatinib can induce mitochondrial membrane potential collapse (Kerkelä et al., 2006).

Another example of interference with SR and mitochondrial homeostasis is cardiotoxicity caused by the multi-kinase inhibitor sunitinib. Sunitinib has been reported to cause ATP depletion in cardiomyocytes through an off-target effect involving AMP-activated protein kinase, or AMPK (Force et al., 2007). The unintentional inhibition of AMPK is thought to activate energy-consuming processes, including protein translation and lipid biosynthesis, which can deplete ATP. Given the tremendous energetic demands of the contracting cardiomyocyte, the improper activation of ATP-consuming processes can be highly toxic (Dyck and Lopaschuk, 2006; Zhang et al., 2008).

### Excitation and contraction

TKI-induced cardiotoxicity can also manifest itself as altered excitation or contraction of cardiac myocytes. These detrimental effects can occur through: (1) direct or indirect modulation of cardiac ionic currents, resulting in pro-arrhythmic electrical activity (Chen et al., 2008; Ghatalia et al., 2015); or (2) structural remodeling that leads to altered myocyte contraction.

TKIs can induce electrophysiological abnormalities directly, via block of ion channels, or indirectly, by altered intracellular signaling that leads to a decrease in K^+^ currents. Because K^+^ currents repolarize the cell membrane during action potentials, either direct or indirect reductions of K^+^ currents can prolong electrocardiographic QT intervals and increase arrhythmia risk. TKIs that are known to block the most relevant K^+^ channel (K_v_11.1), encoded by the gene traditionally known as *hERG* (subsequently renamed *KCNH2*), include crizotinib, sunitinib, and nilotinib. These drugs have been shown to block the channel *in vitro* and to prolong action potentials in human induced pluripotent stem cell-derived cardiomyocytes (hiPSC-CMs) (Doherty et al., 2013). Indirect reductions in K^+^ current may possibly be mediated by Src, a tyrosine kinase that can augment current carried by K_v_11.1 (Schlichter et al., 2014). Thus, dasatinib and bosutinib, which are dual inhibitors of ABL and Src, can potentially cause reduced K^+^ current and QT prolongation (Xu et al., 2009; Gharwan and Groninger, 2015).

Src may also be an important part of the mechanism by which TKIs can induce cellular structural remodeling and impaired contraction. In cardiomyocytes, Src is important for both the organization of sarcomeres and the formation of focal adhesions that connect adjacent cells (Kuramochi et al., 2006). In mice, genetic studies have shown that spontaneous cardiac chamber dilation and disorganization of myofibrils can result from knocking out any of several enzymes in the Src pathway (Peng et al., 2006). Thus, TKIs that inhibit Src may disrupt cardiac contraction by interfering with Src's role in maintaining myocyte structure.

## Complexities of TKI-induced cardiotoxicity and the need for a systems approach

From survival and homeostasis to contractile function, tyrosine kinases perform a wide variety of important roles in the health and function of cardiomyocytes. Although considerable progress has been made to decipher the roles of individual TKs, the breadth of the different mechanisms involved makes it difficult to draw general conclusions about TKI-induced cardiotoxicity. Moreover, even when some mechanistic details have been uncovered, our understanding is primarily qualitative, and biological mechanisms cannot usually be connected to factors such as dosing and the physiological characteristics of individual patients. Given the past success of TKIs as cancer therapeutics and the drive to develop additional TKIs, it would be beneficial to develop a systematic strategy to: (1) evaluate the potential cardiotoxicity of new TKIs; (2) predict the mostly likely mechanisms involved; and (3) suggest strategies for mitigating and/or reversing toxicity. Systems approaches that perform large-scale measurements and quantitatively compare responses to multiple drugs are likely to be extremely useful for understanding the common and distinct features of cardiotoxicity caused by diverse TKIs.

A common approach in systems-level pharmacology studies is to utilize a cell based, high-throughput drug screening assay. Although cardiovascular pharmacology has traditionally not been well-suited for high throughput studies, the development of hiPSC-CMs has expanded the possibilities. For instance, a recent study described the development of a comprehensive assay that evaluated cellular effects of TKIs in hiPSC-CMs (Sharma et al., 2017). Using hiPSC lines from 13 individuals, the investigators examined how 21 FDA-approved small molecule TKIs affected cell viability, contractility, and gene expression. By integrating the results with literature-reported TKI serum levels in patients, the authors developed a novel cardiac safety index for TKIs (Sharma et al., 2017).

Although this study represents a significant milestone in that it integrates cutting edge technologies such as deep sequencing and high-throughput imaging, room for improvement nonetheless remains. Specifically, the experiments performed in this study represent snapshots of cell state after TKI treatment, and the data can therefore provide only limited insight into the dynamics of toxicity development. Moreover, experiments performed using individual drugs cannot predict how either a second drug or a circulating hormone (e.g., adrenaline, angiotensin) might either exacerbate or protect against cardiotoxicity. Although it is of course possible to expand the assay to treat cells with drug pairs and/or add relevant physiological stimuli, it is not clear *a priori* which additional perturbations might be informative or relevant. Finally, even when unambiguous results are seen in cellular high-throughput assays, the mechanistic details often remain hidden. It would therefore be helpful to couple such high-throughput measurements with integrative computational analyses that can potentially overcome these limitations.

## Mechanistic mathematical modeling to improve toxicity testing

One way to address the aforementioned limitations is to use mathematical models that mechanistically describe biological dynamics. When the processes simulated by these models overlap with toxicity mechanisms, the simulations can be used to generate testable predictions, to guide experimental studies, and ultimately to make decisions about new drugs based on a quantitative understanding of benefits and risks. In this context, models that describe biological mechanisms through differential equations are frequently referred to as QSP models (Leil and Bertz, 2014; Gadkar et al., 2016). Although precise definitions remain a matter of debate, QSP models are generally distinguished from both purely empirical, statistical approaches such as computing a risk score for a drug based on a series of measurements (Kramer et al., 2013; Mistry et al., 2015), and pharmacokinetic models that can predict the effects of dosing on cardiotoxicity (van Hasselt et al., 2012) but generally offer only limited mechanistic insight. Although QSP models have been exploited to understand cardiotoxicity caused by anthracyclines (de Oliveira et al., 2016), the application of QSP to TKI-induced cardiotoxicity is still in its early stages. Given the recent development of QSP modeling, it is instructive to consider examples in which mechanistic models have been successfully applied to the study of adverse events, in particular drug-induced liver injury (DILI) (Huang et al., 2013; Shoda et al., 2014; Yang et al., 2015), and drug-induced arrhythmias (Moreno et al., 2011; Sarkar and Sobie, 2011; Britton et al., 2013; Cummins et al., 2014; Grandi and Maleckar, 2016; Yang et al., 2016). Specifically, we emphasize how mechanistic models can be integrated with large *in vitro* data sets, as these studies may provide an important blueprint for future research on cardiotoxicity caused by kinase inhibitors.

An example of the success of QSP models for toxicity applications can be found in the development of DILIsym®, a mathematical model and software package used for predicting DILI. DILIsym® comprises multiple sub-models describing relevant biological processes involved in hepatotoxicity such as drug distribution in the liver, bile acid homeostasis, reactive metabolite generation and disposition, oxidative stress, immune responses, and the hepatocyte life cycle (Woodhead et al., 2017). The value of this approach was recently demonstrated in studies that examined differences in hepatotoxicity between acetaminophen and its less toxic isomer 3′-hydroxyacetnilide. Although the former drug can cause toxicity across many species, the latter has been shown to cause DILI in humans and rats, but not in mice. Using the mechanistic DILIsym® model, a testable hypothesis was generated in which the amount of reactive metabolite produced from each isomer was identified as an important contributor to the observed species differences, and this prediction was confirmed experimentally in a later study (Kyriakides et al., 2016). In addition to the consortium that has developed the DILIsym® package, other groups have gained important insight into DILI through mathematical modeling (Smith et al., 2016; Blais et al., 2017; Thiel et al., 2017).

To understand and predict drug-induced arrhythmias, e.g., Torsades de Pointes (TdP), the Comprehensive *in vitro* Proarrhythmia Assay (CiPA) initiative is highly relevant. This effort, part of the FDA's Critical Path Initiative, aims to improve the accuracy and cost effectiveness of screening for TdP risk. Whereas current *in vitro* methods for predicting TdP risk focus almost exclusively on block of K_v_11.1 (i.e., the *hERG* channel), an approach that is often inadequate, CiPA intends to both assess how drugs block multiple ion channels and to combine these measurements with recordings in hiPSC-CMs and mechanistic simulations (Sager et al., 2014; Fermini et al., 2016). A couple of recently-published studies highlight the value that is gained by utilizing QSP models. Lancaster and Sobie, for example, used models of human ventricular myocytes to simulate physiological changes caused by 67 unique drugs, some that are known to cause TdP, and others that are apparently safe. In addition to providing a classification model that was superior to K_v_11.1 block alone, the simulations provided testable predictions about the most informative assays to perform in cellular experiments and the specific ion transport pathways that, when affected by a drug, may contribute to TdP risk (Lancaster and Sobie, 2016). More recently, Li et al. showed that incorporating the kinetics of K_v_11.1 block into simulations provides superior identification of TdP risk than simply considering steady-state block measurements (i.e., an IC_50_-value), and the study suggested an experimental protocol for measuring drug block kinetics (Li et al., 2017).

The examples of both DILI and drug-induced TdP demonstrate the value that can be added when mechanistic modeling is used to address toxicity. The simulations can uncover the reasons for counterintuitive results, such as drugs that block K_v_11.1 but are nonetheless safe (Kramer et al., 2013; Lancaster and Sobie, 2016; Li et al., 2017), or drugs that only cause hepatotoxicity in some species (Kyriakides et al., 2016; Smith et al., 2016; Blais et al., 2017; Thiel et al., 2017; Woodhead et al., 2017). The simulations can also suggest the prioritization of experiments that are most likely to provide additional insight.

## Initial efforts to use QSP approaches to understand TKI-induced cardiotoxicity

TKI-induced cardiotoxicity is a problem that seems well-suited to a QSP approach because tyrosine kinase signaling encompasses large, complex networks with numerous feedback loops, and understanding how a drug alters TK cascades is therefore extremely complicated. Although mechanistic modeling to predict TKI-induced cardiotoxicity is much less well-developed than for DILI or TdP, efforts that may yield breakthroughs in the next few years are currently underway.

As noted above, an important recent study by Sharma et al. (2017) derived a “toxicity index” by examining effects of TKIs in hiPSC-CMs through several assays. Although the large-scale nature of this study justifies the “systems” label, and the toxicity index is a quantitative risk score, it's important to emphasize that QSP modeling, which can provide mechanistic insight and actionable predictions, is complementary to a high-throughput, data-driven strategy such as that used in this study (Sharma et al., 2017).

Another notable recent effort is a study by Shin et al. (2014). These investigators combined experimental measurements with simulations to uncover mechanisms by which high and sustained doses of the β-adrenergic receptor agonist isoproterenol could increase myocyte susceptibility to apoptosis (Shin et al., 2014). This study is an important example of how simulations often generate novel experimentally-testable predictions, and the work can potentially be extended to examine TKI-induced toxicity.

To fill current gaps in knowledge and obtain new mechanistic insight, the Drug Toxicity Signature Generation (DToxS) Center at Mount Sinai has initiated a large-scale project to advance cellular assays and computational approaches that can improve our understanding of TKI-induced cardiotoxicity. One aspect of this project involves employing QSP models that describe biological processes potentially involved in this cardiotoxicity. We outline here an approach by which whole transcriptome expression assays can be integrated with mechanistic models to classify drugs and generate novel, experimentally-testable predictions.

**Figure 2:**
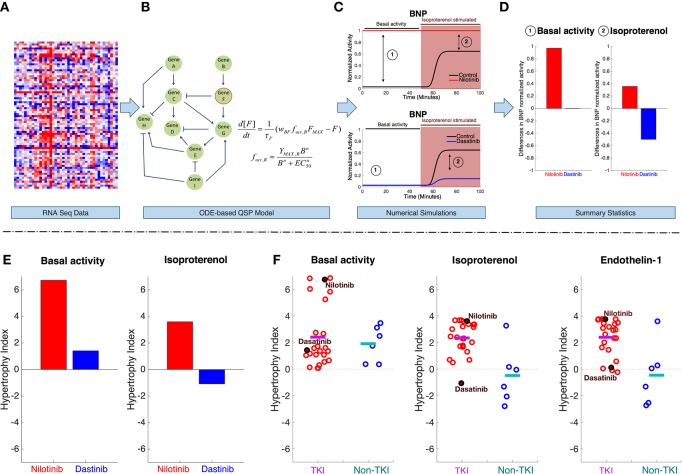
Computational pipeline for integrating gene expression data with QSP models to enable understanding of TKI-induced cardiotoxicity. The pipeline starts with **(A)** mRNAseq data generated from drug treated cells. Using the mRNAseq data, parameters in the QSP model are altered to reflect changes in cell state after 48 h of drug treatment. Specifically, parameters describing maximal activity of model species are scaled based on the changes in gene expression (drug-treated vs. untreated cells). **(B)** The QSP model (Ryall et al., 2012) is composed of ordinary differential equations (ODEs) that describe activation and inactivation of cellular signaling dynamics. Simulations are performed to predict how drug-induced changes in gene expression will influence both basal signaling activity and how cells respond to stimuli. For instance, example simulation results in **(C)** show BNP activity, before, and after stimulation with isoproterenol (a β-adrenergic receptor agonist), in both untreated cells, and cells that have been exposed to two different TKIs for 48 h. These time course simulations predict drug-specific changes, such as an increase in BNP signaling after nilotinib treatment (top) compared with a decrease after dasatinib treatment (bottom). From these time courses, summary statistics **(D)** are collected from steady-state levels of BNP under two conditions (192 basal activity and 193 stimulus). **(E)** Using this pipeline, steady state changes in seven model outputs were computed and summed to generate a metric that we termed the “hypertrophy index.” This provides a summary statistic of the overall hypertrophic risk of a drug under different conditions (e.g., basal activity, left, and isoproterenol stimulation, right). **(F)** Hypertrophy indices computed, under three different conditions, from data obtained in a single cell line after treatment with 24 TKIs, and six non-TKIs (control drugs that are presumed to not cause cardiotoxicity). Each circle represents an individual drug, the line indicates the mean value for each group under basal activity (left), isoproterenol stimulation (middle), and endothelin-1 stimulation (right).

For this part of the analysis, the project has designed a standard experimental protocol (DtoxS—Drug Toxicity Signature Generation Center—SOP, https://martip03.u.hpc.mssm.edu/sop.php) that captures early effects of drugs, as reflected in gene expression changes. The experiments treat cultured cardiomyocyte-derived cells with potentially cardiotoxic TKIs as well as drugs from different classes that are presumably safe. After 48 h, mRNA is harvested, and sequencing is performed to quantify drug-induced changes in gene expression (compared with vehicle-treated controls). Data are released at the DToxS website and can be freely-downloaded (DtoxS—Drug Toxicity Signature Generation Center—Data & Resources, https://martip03.u.hpc.mssm.edu/data.php). The pipeline for integrating these released data with mechanistic mathematical models is shown in Figure 2, top. Changes in mRNA levels in drug treated cells (Figure 2A) can be translated into parameter alterations in models that describe processes potentially relevant to the toxicity (Figure 2B), and simulations are then performed with these models. This workflow assumes that before overt toxicity is induced, drugs can alter the cellular state in relatively subtle ways. Mechanistic simulations may then allow one to predict how this drug-induced altered cellular state influences the response to various physiological stimuli.

For example, results shown here are obtained with a QSP model that describes signaling events relevant to cardiac hypertrophy through a system of 106 ordinary differential equations, each one describing activity of a signaling component (Ryall et al., 2012). This model was chosen for initial simulations because the progression to heart failure due to pathological remodeling often includes a transient induction of hypertrophy. In addition, many known TKI targets (e.g., ERBB2, Raf-1) and critical nodes in cardiac survival signaling (e.g., PI3K, Akt, and ERK) are included. Using this model, simulations were performed to predict how drug-induced network alterations affected 7 hypertrophy biomarkers (the model's “outputs”), under conditions meant to simulate a variety of physiological or pharmacological stimuli (e.g., stretch, angiotensin, EGF, phenylephrine). For instance, time courses in Figure 2C show simulated normalized levels of Brain Natriuretic Peptide (BNP) under six conditions: before and after isoproterenol stimulation, and in three groups of cells: untreated (control), nilotinib-treated, and dasatinib-treated. BNP is an appropriate output to consider because it is both measured in patients with hypertrophy and heart failure and has been shown to be relevant for drug-induced cardiotoxicity (Nousiainen et al., 2002; Sandri et al., 2005; Skovgaard et al., 2014).

These example simulation results, summarized in Figure 2D, suggest that nilotinib leads to increases in BNP, both before and after isoproterenol, whereas dasatinib may reduce the upregulation of BNP that isoproterenol normally causes. The *hypertrophy index*, a summary statistic, condenses results by summing drug-induced changes across seven biomarkers in response to different stimuli applied in the model. The hypertrophy index confirms the impression from the simulated time courses, namely a pro-hypertrophic response to nilotinib contrasted with a slight anti-hypertrophic response to dasatinib (Figure 2E).

By simulating responses to stimuli, using gene expression changes induced by all drugs, patterns begin to emerge. Specifically, in myocytes that are not exposed to a physiological stimulus, TKIs and non-TKIs cause similar changes in hypertrophy biomarkers (Figure 2F). However, simulations predict that when TKI-treated myocytes are also exposed to isoproterenol or endothelin-1, agonists that are used experimentally to induce hypertrophy (Ichikawa et al., 1996; Yamazaki et al., 1996; Shohet et al., 2004; Ryall et al., 2012), the pro-hypertrophic response is exaggerated compared with non-TKI-treated cells.

These preliminary simulation results indicate the potential strengths of combining large scale measurements with mechanism-based mathematical models. First the simulations do not simply describe existing data—they can predict how drug-treated cells will respond to an additional stimulus that has not yet been applied experimentally. These predictions can be subsequently tested. Second, the simulation approach does not merely generate qualitative predictions; because the quantitative models predict that some drugs and/or stimuli may cause large effects whereas others cause only minor effects, this provides a means to prioritize experimental tests and use resources efficiently. Third, when clear differences are observed, for instance between individual drugs or between drug classes, the simulations predict the mechanisms responsible for the differences.

## Future directions

Although the preliminary simulation results shown here are encouraging, they also hint at the future research that must be performed to fully realize the potential of this approach. First, although the simulations predict how drug-induced changes in gene expression may influence both baseline signaling and cellular responses to stimuli, they do not describe direct inhibition of kinase activity by drugs, which is of course the more traditional and straightforward method for simulating drug effects. We excluded these effects from initial simulations because many TKI targets are not included in the model, but future work will expand the model by systematically adding drug targets based on published protein-protein interaction databases (Warde-Farley et al., 2010) and large-scale kinase inhibition assays (Anastassiadis et al., 2011; Davis et al., 2011). For such work, a promising way to expand the model will be to use large-scale, data-driven network identification algorithms (Thiagarajan et al., 2017) that can provide an unbiased approach for identifying potential off-targets.

A second important extension of the work will be to simulate additional biological processes potentially involved in cardiotoxicity. For instance, cell death via apoptosis, which may be important in the development of toxicity caused by some TKIs, has been described mathematically by many previous models (Schleich and Lavrik, 2013; Shin et al., 2014). Once these are tuned to reflect apoptotic signaling in cardiac myocytes, the models can be integrated with the experimental gene expression data to generate novel predictions. Similarly, models describing mitochondrial function, including the production of reactive oxygen species (Aon and Cortassa, 2012; Bazil et al., 2016; Wacquier et al., 2016), and the coupling of electrical excitation, and contractile function (Rice et al., 2008; Tewari et al., 2016), are also likely to be relevant. Finally, once a number of QSP models, describing additional processes, have been added, further secondary analyses can be performed. These include sensitivity analysis to identify the most important nodes in each model (Sobie, 2009), systematic simulations to potential targets for toxicity mitigation or reversal, and effects of combination therapy.

## Conclusions

Here, we have discussed contemporary challenges in understanding TKI-induced cardiotoxicity and have illustrated how a QSP approach can be used to address unresolved questions and improve understanding. Previous successes of QSP in illuminating and predicting other forms of drug toxicity, including hepatotoxicity and drug-induced arrhythmia, demonstrate its potential utility for other drug toxicities. The initial results presented here show how mechanistic models can be integrated with “omics” measurements such as mRNA-seq, generating simulations that can suggest underlying mechanisms and help in prioritizing costly experiments. In the coming years, future work along these lines can be used to develop strategies to mitigate or reverse TKI-induced cardiotoxicity, thereby contributing to the development of therapeutic regimens that are both effective and safe.

## Author contributions

JVS and BC performed an extensive literature review and analysis of published clinical and experimental data. JVS performed simulations and analysis. JVS and ES wrote the initial draft of the manuscript. BC, JvH, MB, and JJS provided critical feedback and editorial suggestions.

### Conflict of interest statement

The authors declare that the research was conducted in the absence of any commercial or financial relationships that could be construed as a potential conflict of interest.
